# Medication work among nonagenarians: a qualitative study of the Newcastle 85+ cohort participants at 97 years old

**DOI:** 10.3399/BJGP.2022.0188

**Published:** 2023-03

**Authors:** Joy Adamson, Helen Hanson, Adam Todd, Rachel Duncan, Barbara Hanratty, Louise Robinson

**Affiliations:** Department of Health Sciences, University of York, York.; Newcastle upon Tyne Hospitals NHS Foundation Trust, Newcastle upon Tyne.; School of Pharmacy, Newcastle University, Newcastle upon Tyne.; Freeman Hospital, Newcastle upon Tyne.; Population Health Sciences Institute, Newcastle University, Newcastle upon Tyne.; Population Health Sciences Institute, Newcastle University, Newcastle upon Tyne.

**Keywords:** Aged, 80 and over, medicines optimisation, polypharmacy, qualitative research, nonagenarians

## Abstract

**Background:**

People aged ≥85 years are the fastest growing section of our population across most high-income countries. A majority live with multiple long-term conditions and frailty, but there is limited understanding of how the associated polypharmacy is experienced by this group.

**Aim:**

To explore the experiences of medication management among nonagenarians and the implications for primary care practice.

**Design and setting:**

Qualitative analysis of medication work in nonagenarians from a purposive sample of survivors of the Newcastle 85+ study (a longitudinal cohort study).

**Method:**

Semi-structured interviews (*n* = 20) were conducted, transcribed verbatim, and analysed using a thematic approach.

**Results:**

In most cases, although considerable work is associated with self-management of medication use, it is generally not experienced as problematic by the older people themselves. Taking medications is habitualised into everyday routines and practices, and is experienced in much the same way as other activities of daily living. For some, the work associated with medications has been relinquished (either partially or wholly) to others, minimising the burden experienced by the individual. Exceptions to this were found when disruptions to these steady states occurred, for example, following a new medical diagnosis with associated medication changes or a major life event.

**Conclusion:**

This study has shown a high level of acceptance of the work associated with medications among this group and trust in the prescribers to provide the most appropriate care. Medicines optimisation should build on this trust and be presented as personalised, evidence-based care.

## INTRODUCTION

People aged ≥85 years are the fastest growing sector of the population in the UK and other high-income countries; the UK population aged ≥85 years is predicted to more than double over the next 20 years, reaching 3.6 million by 2039.^1^ This has important implications for health and social care services, since the majority of those aged ≥95 years live with multiple long-term conditions and the associated polypharmacy.^2^ Taking multiple medications has been found to be associated with a range of negative health outcomes including adverse drug events, drug-interactions, functional decline, cognitive impairment, falls, and urinary incontinence.^3^^–^5 Medicines optimisation is a key feature of health policy, advocating a person-centred approach to safe and effective medicines use.^6^ Central to a medicines optimisation approach is an understanding of how individuals experience and respond to their medications alongside a sense of their desired level of involvement in decision making about their medications.^7^^,^^8^ Several reviews have highlighted important aspects of the patient perspective on medication optimisation including the significance of identity and personal values; individual understanding of medications and morbidity; the physical characteristics of the medications; perceived effects (and adverse effects); the professional/patient relationship; as well as practical aspects such as cost and access to health care. Friends and family have been found to support adherence to medication in practical ways, but their role can be underestimated by health professionals.^9^^–^13

Despite the disproportionate burden of multiple long-term conditions and multiple-medication use faced by older people, the lived experiences of how they incorporate their medications into everyday life are underrepresented in the existing literature. The aim of this study is, therefore, to explore and understand the experiences of medication use among the oldest old (people aged 97 years) to inform medication optimisation practices within primary care service delivery.

## METHOD

An exploratory in-depth qualitative interview study was conducted with a sample of surviving participants from the Newcastle 85+ study. A member of the main study’s public involvement group was involved in the study design, co-developed participant information leaflets, and contributed to the interview topic guide.

**Table:** How this fits in

Accounts of how older people incorporate their medications into everyday life are underrepresented in the existing literature. This study has helped to understand the experiences of medication use among the oldest old (people aged 97 years) to inform medication optimisation practices within primary care service delivery. This study has shown a high level of acceptance of the work associated with medications among this group and trust in the prescribers to provide the most appropriate care. Medicines optimisation should build on this trust and be presented as personalised, evidence-based care.

### Population and recruitment

The Newcastle 85+ study is an observational cohort study of people born in 1921, who reached the age of 85 years during the year of 2006 when recruitment commenced and were registered with a participating general practice in Newcastle upon Tyne or North Tyneside Primary Care Trusts in the UK.^14^ Data were gathered by two methods, a general practice record review and a multidimensional health assessment conducted in the participants’ usual residence by a trained research nurse. A wide variety of information on their health, family and social circumstances, and use of health and care services was collected.^14^ Following baseline assessment, participants were re-assessed at 18, 36, and 60 months, and 10-year follow up at aged 95 years.^15^

This qualitative study purposively sampled from the 80 surviving participants in the 10-year follow up. To reflect the diversity within the cohort and to ensure maximum variation, a sampling frame that included sex (gender), place of residence (living independently, with family members, or residential care), morbidity, and frailty (as captured by a disability measure based on no difficulty with 17 activities of daily living) was used.^14^ Potential participants were contacted via mail and a telephone call to request participation in the study.

### Data collection and analysis

Interviews were conducted between August 2018 and February 2019. Using a semi-structured topic guide (see Supplementary Appendix S1) they explored several aspects of the participants’ day-to-day experiences including their health and health care, social participation, and thoughts about the future. Interviews were conducted by one of the authors, a research nurse with experience of qualitative methods, who had no previous relationship with the participants. All interviews took place in the participants’ own dwelling and lasted approximately 1 hour (ranging from 36 minutes to 2 hours); some participants opted to have a family member present to support them during the interview. Family members contributed to the interviews in practical ways, such as helping with hearing difficulties, filling in details that participants had difficulty remembering, and sometimes adding their own view of the participants’ stories. Informed consent was obtained from all participants following the establishment of capacity.

Interviews were audiorecorded and fully transcribed verbatim. Data were analysed thematically.^16^^,^^17^ A systematic approach was taken, which included: detailed familiarisation; identification and indexing of key themes; and contextualising and interpreting these themes in relation to the broader dataset. Initial coding (carried out by the same author taking the interview) was inductive, whereby coding labels were placed on sections of text. During this process regular meetings were held with another author, a senior qualitative researcher, to discuss codes and themes. Initial findings were presented to a patient representative for comment. This article is based on further development of the inductively identified theme ‘coping with medication’. Further concept development was finalised by the senior qualitative researcher and discussed with the wider research team, drawing on the concept of ‘work’, which has been used within medical sociology to facilitate the understanding of how individuals manage chronic illness.^18^ Constant comparison was conducted, in order to compare data across codes/themes and cases.^19^ In particular ‘negative cases’ were explored; that is, the data from those participants who described an experience of medication management that was different from the majority, enhancing the rigour of the analysis.^19^ Pseudonyms have been given to all participants.

## RESULTS

Interviews were conducted with 20 participants (*n* = 13 female, *n* = 7 male), the majority (*n* = 14) still lived in their own homes and encompassed a range of older people with a range of severity of disability (see Table 1). Eighteen out of 20 participants were taking ≥4 regular medications (ranging from none to ten); one had decided against taking preventative/disease modifying treatment; and the other was using analgesics on an ‘as required’ basis. Five participants were supported during the interview by a family member/carer.

**Table 1.** Participant characteristics

**Participant**	**Sex**	**Disability**	**Housing**
Russell	Male	Moderate	Care home
Pauline	Female	Mild	Owner occupier
Jack	Male	Mild	Owner occupier
Joe	Male	Mild	Owner occupier
Eileen	Female	Mild	Owner occupier
Anne	Female	Severe	Rented home (LA)
Maureen	Female	Moderate	Rented home (private)
Margaret	Female	Severe	Rented home (LA)
Pamela	Female	Mild	Rented home (private)
Malcolm	Male	None	Owner occupier
Bob	Male	Moderate	Care home (LA)
Jean	Female	Moderate	Owner occupier
Mary	Female	Severe	Sheltered housing
Cath	Female	Mild	Owner occupier
Carol	Female	Moderate	Care home
Adele	Female	Mild	Sheltered housing
Penny	Female	Mild	Owner occupier
Tony	Male	Mild	Owner occupier
Alan	Male	Mild	Owner occupier
Angela	Female	Mild	Owner occupier

*LA = Local Authority (an organisation that is officially responsible for all public services and facilities in a particular area).*

### Everyday work involved in medication use

Notable in the participants’ descriptions of their medication use is the degree of work involved in taking multiple medications: from organising the prescription to collecting and storing the medication, knowing the correct dosage times, physically taking the medication, and monitoring for adverse effects. Analysis uncovered three overlapping subthemes relating to different types of work involved including the ‘emotional work’ individuals undertake, relating to how they feel about their medication use; and the ‘cognitive work’ required to understand the reasons for, and effects of, the prescribed medications in the context of their health literacy. Both cognitive and emotional work underpinned the decisions made on accepting or rejecting a medication regimen. This, in turn, governed the ‘instrumental work’ associated with having the right medication available to take at the right time — including ordering and collecting prescriptions, storing, and then physically taking the medications. Yet perhaps surprisingly, for most, this work was not considered a burden:

Participant (P): *‘I’ve got six on the prescriptions, regular prescriptions. Apart from those, which are prescribed by the medical group, I take little … What do you call them? … Extra tablets that are my own choice. So there would be about 10 probably.’*

Interviewer (I): *‘How do you feel about taking that number of tablets?’*

P: *‘Of the six on the prescription, I think there are about four — at least — that I’ve had to take all my life for various … I’ve got anaemia, pernicious anaemia. Yes, yes. So I’ve got to take little things like that. It doesn’t bother me … No trouble at all, no.’* (Joe, Male [M])

It would appear there is an apparent paradox between the work involved in medications management and the associated burden, however, this can be explained in terms of the degree to which, over time, medication use has become accepted, adhered to, and habitualised.

### Habitualised management

The majority of participants had long since accepted the need for prescribed medications in a matter-of-fact way, without evidence of actively engaging with any significant decision making around this on a day-to-day basis. To a general question about their day so far, one participant responded:

*‘Well I got up this morning, had a shower, got dressed and then I came through and had some toast and marmalade, I took my tablets, washed my breakfast things, put my little bit of washing out, run the sweeper over the floor and that was it.’*

(Maureen, Female [F])

This was a typical answer, suggesting that for many participants that were interviewed, medication use is an accepted and firmly embedded part of their autonomous daily routine, associated with minimal burden. One ubiquitous element in the descriptions of instrumental work was the presence of routines, whether these are daily, weekly, or monthly. The weekly visit to the pharmacy on a Friday was a fixed point in this participant’s routine:

*‘Hair tomorrow, I go and get my hair done tomorrow, 9.30 am, then tablets on Friday. I go up for them to the chemist. They could deliver it, but I said, “Well, I never know when I’m gonna be in.” So, I’d rather go up for them myself. So, I get them on a Friday, when I get some ham or something and some rolls for when* [daughter’s name] *comes Saturday and we have our lunch.’*

(Mary, F)

Various strategies to optimise adherence were described, and demonstrated medication use was completely entrenched into daily routine practices reflecting that, for many participants, their lives were more generally characterised by domestic routines. For example, some placed medication in different locations around the house to be at hand when needed:

*‘I’m usually setting my breakfast up about 8.00 am, and the washing up and putting the next day’s medication in the two little containers, one I take up to bed for the morning’s medication, and the night-time one I keep in there.’*

(Tony, M)

The importance of routine was reaffirmed when participants described lapses in adherence were most often associated with changes in their normal pattern of activity:

*‘Say for instance on Sunday night there. I forgot to take them. No, Saturday night it must have been. With my son. We had been out all day and came in late at night. About 10.00 pm it was when we came in. I was so eager getting undressed and getting into bed after having a wash that I forgot to take my tablets. It’s just occasions like that when I might I forget you see.’*

(Jack, M)

For most participants much of the cognitive and emotional work involved in decision making relating to medication adherence had taken place in the past and generally no longer required further processing on a day-to-day basis. This is not to imply that participants were necessarily passive in their interest or understanding of medications. While this varied, some indicated a good medication literacy:

*‘There are not so many now but the important, the immune system one, MMF* [mycophenolate mofetil]*, I used to be on 250 mcg capsules morning and evening, and then* [name of doctor] *after tests, the antibodies that had been causing the encephalitis, I was having these encephalitis fits … Then they found 10 times the normal amount of this particular antibody so they put is* [me] *on that MMF.’*

(Tony, M)

Many participants had taken medications (sometimes the same medications) for years and even in the absence of direct evidence of effectiveness, many of those interviewed were still strongly committed to taking their prescribed medications regularly. The motivation for ongoing medication adherence most commonly articulated was a trust in the medical professional prescribing the medication:

I: *‘You feel as though they’re helping you, the medications?’*

P: *‘Well, they’re bound to be. They’re bound to be,* [interviewer’s name]*, or I wouldn’t be getting them.’* (Jean, F)

This trust appeared to be based on a combination of the stated accepted expertise of the professionals (based on the symbolic capital of the prescribing clinicians), but also indicated implicitly by the lack of any adverse events or side effects mentioned expressly in the accounts:

*‘It doesn’t worry me, because it’s only three things and I know it’s not doing me any harm. It’s for my own good. I just swallow them, full stop. I don’t even think. I just take them and get on with it.’*

(Jean, F)

Only a few participants described occasional incidents of swallowing problems and adverse effects.

While the majority of the responders had reached a steady state in their medication management, this was not to suggest that no cognitive or emotional work was being undertaken. For most, there was still an implicit monitoring of effectiveness or side effects from medications by the participants. Some were content that their medications were beneficial, often citing their own longevity as proof, however, this seemed to vary by medication class and the availability of evidence on which to base these perceptions. For example, medication for analgesia was given as an example, where effectiveness could be easily monitored:

*‘My taking the codeine and paracetamol, it doesn’t make things completely back to normal, but I think it helps. The rest of them, I probably don’t notice the difference. I know my blood pressure is normal, so I know the tablets are controlling that.’*

(Cath, F)

I: *‘Is there anything that you are able to do to ease the pain or to make your knees better?’*

P: *‘I have ointments, but nothing seems to do any good. And I take ibuprofen tablets, but they don’t seem to help much there.’* (Margaret, F)

For longstanding difficulties associated with particular medication use, participants articulated the autonomous solutions they had derived in order to achieve an acceptable balance between the potential benefits of the medication and the negative impacts of adherence:

*‘Occasionally I drop them off, but then I’m probably advised to restart them. I mean, the one for osteoporosis, the alendronic acid one, anybody who takes it will tell you, it’s a real misery. You aren’t supposed to lie down, or have anything to eat for half an hour before. The nurse might say, take it in the night, but you don’t want to be sitting up in the early morning, or the middle of the night. It really is a nuisance to take. The rest of the tablets you just take them and that’s that.’*

(Cath, F)

Participants appeared to derive comfort from the familiar routines and satisfaction from performing these routines competently, this role fulfilment may help to foster a positive sense of identity as well as demonstrating (and protecting) their autonomy, especially for participants who were growing more dependent in other areas of their lives.

For this group of the oldest old, as would be expected, previous practices relating to medication management had been challenging for some due to physical and/or cognitive decline. This meant relinquishing some of their autonomy over the maintenance of their health and had to call on their social capital to fulfil the task of medication management:

I: *‘Are there things that are difficult for you nowadays to go on holiday?’*

P: *‘Oh, yes. It’s awkward for medication.* [Daughter’s name]’*s used to it but I get quite mixed up with that, checking it before and keeping it in order while we’re away.’* (Tony, M)

### Diminishing autonomy

The process of relinquishing autonomy is slow, adjusting to physical and cognitive change occurs over time and new routine practices are gradually formed seeking assistance across a range of activities of daily living, in a stepwise fashion. In terms of medications, in some cases elements of the instrumental work associated with medicines was shared or relinquished to members of the participants social network, diminishing the potential burden experienced:

P: *‘I sit at the table, and* [daughter’s name] *brings them. She helps me, now, to get the blooming things out. I sit and do 4 weeks at a time.’*

Daughter: *‘They’re all written down. She follows the names. I bring all the boxes, the Tupperware boxes out, and then she puts them all in and does them for … She sits and does them.’* (Eileen, F)

Doctors’ surgeries and pharmacies typically offer multiple means of communication to order repeat prescriptions, including in person, telephone, and internet. Nevertheless, a combination of sight, hearing, and mobility impairments made accessing these services difficult for some, and family, friends, or paid carers were supporting this work.

In the most extreme cases, participants did not describe any burden associated with medication management as they had relinquished responsibility for the mainstay of the associated work into either informal or formal support networks. For example, one participant no longer knew the names of any of her medications or the reasons for why she took them. She was living in her own home with carers attending four times a day to assist her with personal care, meals, and medications:

P: *‘Well, to tell you the truth, I don’t know what I take.’* [laughter]

I: *‘Who looks after your medicines, then?’*

P: *‘Oh, the girls.’* (Pauline, F)

This participant was still committed to taking her medications and did so regularly, with the assistance of her care workers. Doing less of the instrumental work associated with medications appeared to be closely associated with a lack of cognitive or emotional work including knowing which drug they were taking and why, or feeling anxious about their medication use. This also tended to happen in a context where participants had given up or were giving up responsibility for other areas of their life into the trusted domain of professionals and/or family members.

### Disrupted management

There were two clear exceptions within the sample, who fell outside of ‘habitual management’ having suffered a disruption that had led to a change in their medication management practices. The first example was a woman with a new health problem struggling to adjust. She described her medications as:

*‘Bane of me life.’*

(Pamela, F)

She was still taking the medications, but she had experienced some severe adverse effects and was anxious that the doses were still not correct. Being outside of the ‘steady state’ where greater cognitive and emotional work were implied in order to understand the change in health status and the consequences of this in terms of medicines management, were associated with much greater feelings of burden.

The second exception was a man who had stopped all medications, no longer visited his GP, and had refused investigations of a skin lesion. He described his choice, saying:

*‘I decided I would live with it, until my time on this planet was up.’*

(Bob, M)

This rejection of medication and other forms of medical intervention have been a recent change in his outlook, following a major disruptive life event — the death of his wife — and he feels he no longer wants to prolong his own life, taking the autonomous decision to cease his medication use alongside other forms of health care.

## DISCUSSION

### Summary

This study, which has explored medication work among nonagenarians, found that in most cases, although medication use requires emotional, cognitive, and instrumental work it is generally not experienced as problematic. Medication use is habitualised into everyday routines and practices, like ‘having toast and marmalade’, and is regarded in much the same way as other activities of daily living.

Following (what can be) years of using medication for a specific indication, most issues associated with perceived effectiveness or adverse effects have been addressed and routines have been established. These older people take an autonomous role in choosing to adhere to medication regimens and practical ways of realising this aim. For some, the work associated with medications has been relinquished (either partially or wholly) alongside their diminishing autonomy, minimising the burden experienced by the individual. Exceptions to this were found when disruptions to these steady states occurred, for example, following a new medical diagnosis with associated medication changes or a major life event.

### Strengths and limitations

The study presents a rare opportunity to understand the experiences of nonagenarians. Narratives relating to the everyday lives of participants were generated through the interview process; therefore, medications were talked about as part of these accounts, rather than in answer to specific questions relating to particular medications. As such, the data were able to show the significance of medication work as part of their lives as a whole and capture their implicit views on medications and how they coped with these. However, a lack of direct questioning may have limited findings with regards to medicines efficacy and adverse effects in some cases.

All of those interviewed were longstanding participants in the Newcastle 85+ cohort study and, as such, were all from a small area of the UK, White British, and had shown a willing commitment to participation in research. This may have limited the range of accounts that were obtained and hence transferability to other populations. It is also important to note that findings may be subject to a cohort effect and may not have transferability to future generations when they reach this age range.

Participants were offered the opportunity to have someone present at the interviews — five interviews were conducted with the participant and a carer. It is possible that the participant provided a different account than they would have unaccompanied. However, this is unlikely to have changed the main messages of the study.

### Comparison with existing literature

Several of the points raised in this study echo findings from previous research, for example, daily routines being used as memory aids and that forgetting medication was worsened when routines were disturbed; being diagnosed with a life-limiting illness impacts on perceived value of certain medications; the use of physical medication organisation systems (for example, dosette boxes) to assist with the management of complex medication use; and the importance of social support.^20^^–^27

However, there are also features from the previous literature that were largely absent from the accounts generated in this study. For example, studies have indicated a range of instrumental difficulties experienced by individuals taking multiple medications, including: the collection of medications, obtaining repeat prescriptions, and the cost of medications. These issues did not feature in this current research, possibly explained by the older age of the sample and the policy context within which the data were collected. The research took place in the UK, therefore all prescriptions for those aged >60 years were free of charge. Most pharmacies have services in place to assist with repeat prescriptions and have home delivery options on request. The participants in this study had established routines of performing this work themselves, or with the help of formal or informal support. Likewise, there was little mention of adverse effects, swallowing difficulties, and medication volume, which have been highlighted in previous work.^20^^–^24 Again, it is possible that such issues may have been addressed earlier in the individual’s ‘medications career’ and were, therefore, no longer a significant issue for participants who were in a steady state of medication management.

Studies including younger populations often highlight the burden felt by participants when medication use interferes with social interactions and other daily activities;^20^^,^^21^^,^^25^^–^27 however, this also did not feature heavily in the accounts of the current study’s participants. This may, in part, be due to fewer competing priorities within this population sample of the nonagenarians, and that medication work was seen as integral to everyday activities, rather than in conflict with it. It is likely that identity work (that is, any modifications to an individual’s identity) resulting from the onset of chronic illness and associated medication use has been completed by this group, having an established acceptance of any physical and mental decline experienced by the age of 97 years. Being able to cope with or adjust to a complex medication regimen signified a personal success and can produce feelings of self-worth and, by extension, senses of health and wellbeing.^22^

### Importance for research and practice

As stated above, the data presented here was generated through qualitative interviews intended to explore participants’ day-to-day experiences. Medication optimisation was not the sole focus of the interviews. Further research would benefit from considering more specifically, and in more detail, the interaction between older people, their specific (multiple) medications, and their primary care practitioners. This could address important issues relating to medication reviews, the appropriateness of medications, and safe autonomy relating to medication practices at home.

Findings from this study provide insights that may be of help to clinicians in approaching patient-centred discussions with those from the oldest old cohort, through a greater understanding of the range of experiences they are likely to encounter among this population group. The study found that, for many participants in this cohort, there was an unquestioning acceptance of medications and a great deal of trust in prescribing clinicians. Policy documentation has been lacking in guidance for clinicians around helping these supporting parties manage regimens on behalf of older people. It is important to note that for this age group the greatest burden from medication use happens when a disruption occurs; support in terms of information provision, regular review, and follow-up should be provided to minimise the impact of disruptions.

Given the international focus of deprescribing and reducing inappropriate polypharmacy^28^ this study has several important policy implications. Structured medication reviews are incorporated into routine clinical practice, with many pharmacists now specifically employed to optimise medications in older people. This study’s findings suggest that medication reviews should be tailored to the needs of the individual, and a standardised approach for older people may not be appropriate, especially among nonagenarians. The desired level of patient involvement in decision making about medications should also be acknowledged. Indeed, any approach to reduce inappropriate medication in older people should focus on their clinical and physical, as well as their social and psychological, needs.^29^ Tools such as STOPP/START,^30^ which are used to identify potential inappropriate medication in older people, are helpful in this regard, but should not be solely used as a way to reduce or stop medication. This study has shown that participants continued to take their medication because they thought they were ‘bound to be’ helping. The timing of medication review for this cohort may also be important: in addition to having a medication review over a defined time period, a medication review could also be triggered by any significant change in prescribing (for example, initiating a new medication) or life event for the patient.

Central to the success of a shared decision-making approach is an understanding of how individuals experience and respond to their medications alongside a sense of their desired level of involvement in decision making about their medications. This study has provided insights into how nonagenarians interact with their medications. It has shown a high level of acceptance of the work of medication management among this group and trust in the prescribers to provide the most appropriate care. This suggests that any rationalisation of medications must build on this trust and be presented as personalised, evidence-based care.
